# Characterization of the Trimethylamine *N*-Oxide Transporter From *Pelagibacter* Strain HTCC1062 Reveals Its Oligotrophic Niche Adaption

**DOI:** 10.3389/fmicb.2022.838608

**Published:** 2022-02-28

**Authors:** Chao Gao, Nan Zhang, Xiao-Yan He, Ning Wang, Xi-Ying Zhang, Peng Wang, Xiu-Lan Chen, Yu-Zhong Zhang, Jun-Mei Ding, Chun-Yang Li

**Affiliations:** ^1^State Key Laboratory of Microbial Technology, Marine Biotechnology Research Center, Shandong University, Qingdao, China; ^2^College of Marine Life Sciences, Frontiers Science Center for Deep Ocean Multispheres and Earth System, Ocean University of China, Qingdao, China; ^3^Laboratory for Marine Biology and Biotechnology, Pilot National Laboratory for Marine Science and Technology, Qingdao, China; ^4^School of Bioengineering, Qilu University of Technology, Jinan, China; ^5^Engineering Research Center of Sustainable Development and Utilization of Biomass Energy, Ministry of Education, Yunnan Normal University, Kunming, China

**Keywords:** TMAO, ABC transporter, substrate binding protein, SAR11 bacteria, niche adaptation

## Abstract

Trimethylamine *N*-oxide (TMAO), which was detected at nanomolar concentrations in surface seawaters, is an important carbon, nitrogen and/or energy source for marine bacteria. It can be metabolized by marine bacteria into volatile methylated amines, the second largest source of nitrogen after N_2_ gas in the oceans. The SAR11 bacteria are the most abundant oligotrophic plankton in the oceans, which represents approximately 30% of the bacterial cells in marine surface waters. Genomic analysis suggested that most SAR11 bacteria possess an ATP-binding cassette transporter TmoXWV that may be responsible for importing TMAO. However, it was still unclear whether SAR11 bacteria can utilize TMAO as the sole nitrogen source and how they import TMAO. Here, our results showed that *Pelagibacter* strain HTCC1062, a SAR11 bacterium, can grow with TMAO as the sole nitrogen source. TmoXWV from strain HTCC1062 (TmoXWV_1062_) was verified to be a functional TMAO importer. Furthermore, TmoX_1062_, the periplasmic substrate binding protein of TmoXWV_1062_, was shown to have high binding affinities toward TMAO at 4°C (*K*_*d*_ = 920 nM), 10°C (*K*_*d*_ = 500 nM) and 25°C (*K*_*d*_ = 520 nM). The high TMAO binding affinity and strong temperature adaptability of TmoX_1062_ reveal a possible oligotrophic niche adaptation strategy of strain HTCC1062, which may help it gain a competitive advantage over other bacteria. Structure comparison and mutational analysis indicated that the TMAO binding mechanism of TmoX_1062_ may have differences from the previously reported mechanism of TmoX of *Ruegeria pomeroyi* DSS-3. This study provides new insight into TMAO utilization by the widespread SAR11 bacteria.

## Introduction

Marine phytoplankton generate approximate one-half of the global primary production in the oceans, with a large fraction turning into dissolved organic matter (DOM) by various mechanisms (Falkowski et al., 1998; Azam and Malfatti, 2007). Trimethylamine *N*-oxide (TMAO) is an important component of marine DOM and a compatible osmolyte for a variety of marine biota (Gibb and Hatton, 2004; Carpenter et al., 2012). It is also a nitrogen and/or carbon source for marine heterotrophic bacteria (Lidbury et al., 2015). The concentrations of TMAO range from low nanomolar (nM) in coastal and open ocean surface waters to low micromolar (μM) in deep sea (Gibb et al., 1999; Gibb and Hatton, 2004). TMAO participates in various physiological processes in marine organisms (Seibel and Walsh, 2002). In deep-sea organisms, TMAO can act as a potent protein stabilizer, playing a central role in counteracting the protein-denaturing effect of urea (Ma et al., 2014; Liao et al., 2017; Ganguly et al., 2020). TMAO can also serve as a piezolyte, which can be accumulated in bacteria and fish to improve the survival of organisms at high hydrostatic pressure (Yancey et al., 2014; Yin et al., 2018; Qin et al., 2021). Furthermore, TMAO can be catabolized by marine bacteria to small, volatile, methylated amines (MAs), such as trimethylamine (TMA), dimethylamine (DMA) and monomethylamine (MMA), which are precursors of the greenhouse gas nitrous oxide (Dos Santos et al., 1998; Lidbury et al., 2017).

**FIGURE 1:**
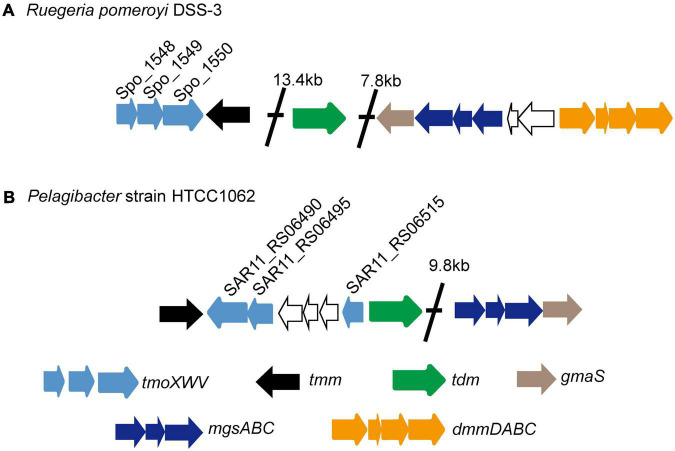
The gene clusters involved in TMAO transport and metabolism in the MRC bacterium *R. pomeroyi* DSS-3 **(A)** and in the SAR11 bacterium HTCC1062 **(B)**. Tdm, trimethylamine N-oxide demethylase; Tmm, trimethylamine monooxygenase; TmoXVW, ATP-dependent TMAO transporter; DmmDABC, DMA monooxygenase; MgsABC, N-methylglutamate synthase; GmaS, γ-glutamylmethylamide synthetase.

SAR11 bacteria are the most abundant oligotrophic bacteria in ocean surface waters, and play an important role in mineralizing marine DOM (Morris et al., 2002). *Pelagibacter* strain HTCC1062, the first cultivable SAR11 bacterium, can utilize TMAO to generate ATP (Giovannoni et al., 2005; Sun et al., 2011). The marine *Roseobacter* clade (MRC) bacterium *Ruegeria pomeroyi* DSS-3, which can grow with TMAO as the sole nitrogen source, can also utilize TMAO to produce intracellular ATP (Lidbury et al., 2015). The gene cluster encoding proteins for TMAO transport and metabolism has been identified in *R. pomeroyi* DSS-3 (Figure 1A; Lidbury et al., 2014, 2015, 2017). In strain DSS-3, TMAO can either be imported from marine environment by TmoXWV, an ATP-binding cassette (ABC) importer specific for TMAO, or be converted *in vivo* from TMA through the catalysis of TMA monooxygenase Tmm (Chen et al., 2011; Lidbury et al., 2014; Li et al., 2017). Then, TMAO in the cells is catabolized to DMA by TMAO demethylase Tdm (Lidbury et al., 2014; Sun et al., 2019). With the catalysis of DMA monooxygenase DmmDABC, DMA is further catabolized to MMA (Lidbury et al., 2017; Sun et al., 2019), which can be converted to γ-glutamylmethylamide by γ-glutamylmethylamide synthetase GmaS (Chen Y. et al., 2010; Wischer et al., 2015; Wang et al., 2021). Bioinformatic analysis indicated that *tmoXWV* homologs are prevalent in SAR11 bacteria (Lidbury et al., 2014). However, genomic analysis suggested that SAR11 bacteria lack *dmmDABC* (Figure 1B; Lidbury et al., 2017), which is essential for TMAO utilization as the nitrogen source in *R. pomeroyi* DSS-3. So far, it is still unknown whether SAR11 bacteria can utilize TMAO as a nitrogen source.

In the TMAO transporter TmoXWV, TmoX is the periplasmic TMAO binding protein, the TMAO binding mechanism of which in *R. pomeroyi* DSS-3 has been revealed on the basis of structural and biochemical analyses (Li et al., 2015). Phylogenetic analysis indicated that TmoXWV belongs to the glycine betaine/proline betaine-type ABC transporter family, and TmoX belongs to the cluster F III of the ABC transporter superfamily (Lidbury et al., 2014). Cluster F III consists of substrate binding proteins specific for different compatible osmolytes, including betaine, carnitine, choline and TMAO (Berntsson et al., 2010; Lidbury et al., 2014; Rice et al., 2014; Beis, 2015). It has been found that TmoX homologs from MRC and those from SAR11 bacteria form two different branches in the phylogenetic tree (Lidbury et al., 2014). Therefore, the TMAO binding mechanism of SAR11 TmoX may have differences from that of *R. pomeroyi* DSS-3 TmoX.

This study aimed to investigate whether SAR11 bacteria can utilize TMAO as a nitrogen source and how they import TMAO with strain HTCC1062 as a model. We found that strain HTCC1062 can grow with TMAO as the sole nitrogen source. Genetic work demonstrated that TmoXWV_1062_, the TmoXWV homolog in HTCC1062, is a functional TMAO importer. TmoX_1062_, the periplasmic substrate binding protein of TmoXWV_1062_, was shown to have high binding affinities toward TMAO at 4–25°C by biochemical studies. The TMAO binding mechanism of TmoX_1062_ was further analyzed by structural modeling and mutational analysis.

## Materials and Methods

### Bacterial Strains and Growth Conditions

Strain HTCC1062 was cultured in AMS1 medium amended with 25 μM glycine, 10 μM methionine and 50 μM pyruvate at 16°C according to the reported protocol (Rappe et al., 2002; Tripp, 2013). AMS1 was sparged with CO_2_ for 5 h followed by sparging with air for 10 h. The pH of the resulting AMS1 typically ranged from 7.5 to 7.7. Cells of strain HTCC1062 were stained with SYBR Green I (Molecular Probes, America) and counted with a Guava Technologies flow cytometer (Millipore, America). The *E. coli* strains DH5α, BL21(DE3) and WM3064 were grown in the Lysogeny Broth (LB) medium at 37°C. Diaminopimelic acid (0.3 mM) was added into the LB medium to culture *E. coli* WM3064. *R. pomeroyi* DSS-3 was purchased from the Leibniz Institute DSMZ-German Collection of Microorganisms and Cell Cultures and was cultured in 974 medium at 30°C according to the protocol provided^1^.

### Real-Time qPCR Analysis

Strain HTCC1062 was firstly cultured in AMS1 medium amended with 25 μM glycine, 10 μM methionine and 50 μM pyruvate. When the concentration of cells reached 2 × 10^7^ cells/ml, TMAO was added into the medium with a final concentration of 0.8 mM. The group without the addition of TMAO was set up as a control. After 0.5 or 2 h incubation, RNA was extracted from the cells using the RNeasy mini kit (Qiagen, America), and was subsequently reverse-transcribed to cDNA using Goldenstar™RT6 cDNA Synthesis Kit (TsingKe, China). The qPCR experiments were performed using a Light Cycler II 480 System (Roche, Switzerland) following the instructions of SYBR^®^ Premix Ex TaqTM (TaKaRa, Japan) with the following cycling conditions: 95°C for 5 min, 45 cycles of 95°C for 10 s and 60°C for 30 s. The *recA* gene was used as an internal reference gene.

### Genetic Manipulations

Deletion of the *tmoW* gene of *R. pomeroyi* DSS-3 was performed by pK18*mobsacB*-Ery based homolog recombination (Wang et al., 2015). The upstream and downstream sequences of the *tmoW* gene were amplified with primer sets *tmoW*-UP-F/*tmoW*-UP-R and *tmoW*-Down-F/*tmoW*-Down-R (Supplementary Table 1 and Supplementary Figure 1). Then, the PCR fragments were inserted to the vector pK18*mobsacB*-Ery with *Hin*dIII/*BamH*I as the restriction sites to generate pK18Ery-*tmoW*, which was transferred into *E. coli* WM3064. Next, the plasmid pK18Ery-*tmoW* was mobilized into *R. pomeroyi* DSS-3 by intergeneric conjugation with *E. coli* WM3064. To select for colonies in which the pK18Ery-*tmoW* had integrated into the *R. pomeroyi* DSS-3 genome by a single crossover event, cells were plated on the marine 2,216 agar plates containing erythromycin (25 μg/ml). Subsequently, the resultant mutant was cultured in the marine broth 2,216 medium and plated on the marine 2,216 agar plates containing 10% (w/v) sucrose to select for colonies in which the second recombination event occurred. For complementation of the Δ*tmoW* mutant, the *tmoXWV*_1062_ gene cluster with its native promoter was amplified from the genomic DNA of HTCC1062 with primer sets *tmoXWV*_1062_-350Up-F/*tmoXWV*_1062_-Down-R (Supplementary Table 1). The PCR fragments were digested with *BamH*I and *EcoR*I, and then inserted into the vector pHG101 to generate pHG101-*tmoXWV*_1062_. This plasmid was then transformed into *E. coli* WM3064, and mobilized into the Δ*tmoW* mutant of *R. pomeroyi* DSS-3 by conjugation.

### Gene Cloning, Point Mutation, and Protein Expression and Purification

The full-length *tmoX*_1062_ gene was amplified from the genomic DNA of HTCC1062 by PCR using *FastPfu* DNA polymerase (TransGen Biotech, China), and was subcloned into the *Nde*I/*Xho*I restriction sites of the pET22b (Novagen, America) vector with a C-terminal His-tag. All of the point mutations in *tmoX*_1062_ were performed with the QuikChange^®^ mutagenesis kit II (Agilent, America). The wild-type (WT) TmoX_1062_ protein and all of the mutants were expressed in *E. coli* strain BL21(DE3). The recombinant *E. coli* strains were cultured at 37°C in LB medium to an OD_600_ of 0.8–1.0 and then incubated at 16°C for 16 h with 0.5 mM isopropyl β-D-1-thiogalactopyranoside (IPTG) as an inducer for recombinant protein expression. The recombinant proteins were purified first with Ni-affinity column (GE Healthcare, America), and then with gel filtration on a Superdex-75 column (GE Healthcare, America) eluted with the buffer containing 10 mM Tris–HCl (pH 8.0) and 100 mM NaCl. Approximately 2 mg recombinant TmoX_1062_ protein was obtained from 1 liter of culture.

### Isothermal Titration Calorimetry Measurements

Isothermal titration calorimetry (ITC) measurements were performed using a PEAQ-ITC system (Malvern, Britain). The sample cell was loaded with 250 μl of protein sample (30 μM), and the reference cell contained distilled water. The syringe was filled with 70 μl of TMAO (200 μM). The proteins and TMAO were kept in the same buffer containing 10 mM Tris–HCl (pH 8.0) and 100 mM NaCl. Titrations were carried out by adding 0.4 μl of TMAO for the first injection and 1.5 μl for the following 12 injections, with stirring at 750 rpm/min.

### Circular-Dichroism Spectroscopic Assays

Wild-type TmoX_1062_ and all of the mutants were subjected to circular-dichroism (CD) spectroscopic assays at 20°C on a J-1500 spectropolarimeter (Jasco, Japan). CD spectra of the samples at a final concentration of approximately 10 μM were collected from 250 nm to 200 nm at a scan speed of 200 nm/min with a bandwidth of 1 nm. All of the samples were in a buffer containing 10 mM Tris–HCl (pH 8.0) and 100 mM NaCl. To determine the *T*_m_ of TmoX_1062_, the temperature was raised from 20 to 80°C in 1 h.

## Results and Discussion

### *Pelagibacter* Strain HTCC1062 Can Grow With Trimethylamine *N*-Oxide as the Sole Nitrogen Source

**FIGURE 2:**
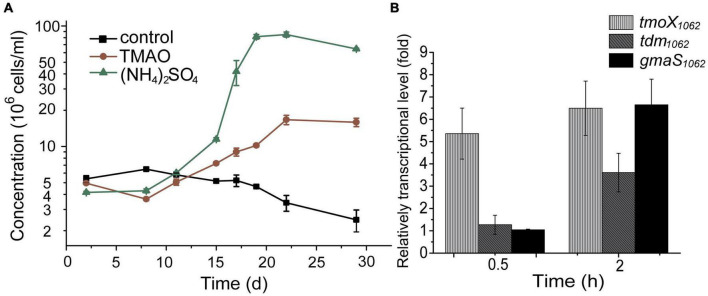
TMAO utilization by strain HTCC1062. **(A)** The growth curves of HTCC1062 with 0.8 mM TMAO or 0.4 mM (NH_4_)_2_SO_4_ as the sole nitrogen source. DMSP was used as the sulfur source. Bacterial cells cultured without nitrogen source were used as the control. The concentration of bacteria was determined by flow cytometry. **(B)** RT-qPCR assay of the transcriptions of genes possibly involved in the TMAO transport and metabolism in HTCC1062. The bacterium cultured without TMAO in the same medium was used as the control. The *recA* gene was used as an internal reference gene. The error bar represents standard deviation of triplicate experiments.

To investigate whether strain HTCC1062 can grow with TMAO as a nitrogen source, we replaced (NH_4_)_2_SO_4_ in AMS1 medium by TMAO. Methionine, which is usually used as the reduced sulfur, was also replaced by dimethylsulfoniopropionate (DMSP) to avoid the possible interference of its nitrogen atom. As shown in Figure 2A, strain HTCC1062 showed noticeable growth in the medium with TMAO as the sole nitrogen source, although its growth on TMAO was much weaker compared to that on (NH_4_)_2_SO_4_. This result suggests that strain HTCC1062 should contain TMAO transporter and enzymes involved in TMAO metabolism.

In *R. pomeroyi* DSS-3, TMAO can be transported into the cell through a TMAO specific transporter TmoXWV (Lidbury et al., 2014), and is then utilized as a nitrogen and energy source with the catalysis of several enzymes, including Tdm, DmmDABC and GmaS (Lidbury et al., 2015). Genomic analysis suggests that strain HTCC1062 possesses *tmoXWV*, *tdm* and *gmaS* homologs (*tmoXWV*_1062_, *tdm*_1062_ and *gmaS*_1062_, respectively). However, no *dmmDABC* homolog was identified from the genome of strain HTCC1062 (Lidbury et al., 2017; Figure 1B). RT-qPCR analysis showed that the transcriptions of *tmoX*_1062_, *tdm*_1062_ and *gmaS*_1062_ were all up-regulated by TMAO (Figure 2B), suggesting that these genes may be functional in TMAO import and metabolism. Thus, the SAR11 bacterial strain HTCC1062 may import and metabolize TMAO *via* a pathway generally similar to that of the MRC bacterial strain DSS-3, except that strain HTCC1062 may recruit an isoenzyme of DmmDABC to convert DMA to MMA. Next, we characterized TmoXWV_1062_ of strain HTCC1062 to investigate how SAR11 bacteria import TMAO in this study.

### Functional Analysis of TmoXWV in HTCC1062

**FIGURE 3:**
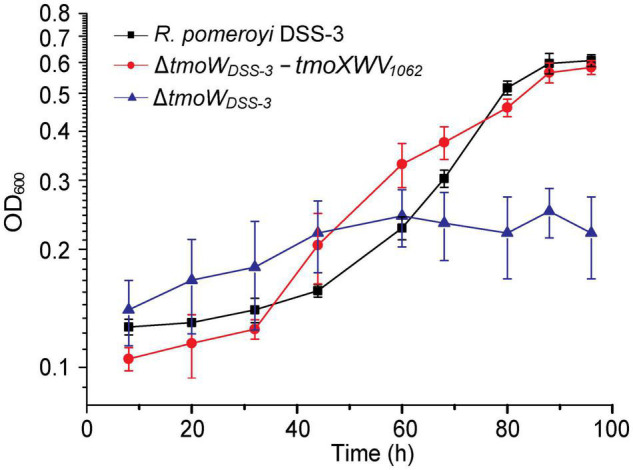
Growth curves of the WT *R. pomeroyi* DSS-3, the Δ*tmoW*_*DSS–3*_ mutant, and the complemented mutant Δ*tmoW*_*DSS–3*_-*tmoXWV*_1062_. All strains were cultivated with TMAO (2 mM) as the sole nitrogen source. The error bar represents standard deviation of triplicate experiments.

It has been reported that the *tmoW-*deleted mutation in *R. pomeroyi* DSS-3 disables its capacity to grow with TMAO as the sole nitrogen source (Lidbury et al., 2014). TmoXWV_1062_ of strain HTCC1062 shares ∼41% sequence identity to the functional TmoXWV of *R. pomeryi* DSS-3. Because currently genetic manipulation cannot be performed in SAR11 bacteria, we tried to demonstrate the TMAO-importing function of TmoXWV_1062_ in a *tmoW-*deleted mutant of *R. pomeroyi* DSS-3. We constructed the mutant Δ*tmoW*_*DSS–3*_ by deleting the majority of gene *tmoW* from the *R. pomeroyi* DSS-3 genome, and then complemented this mutant with *tmoXWV*_1062_ to generate the complemented strain Δ*tmoW*_*DSS–3*_-*tmoXWV*_1062_ that contains the *tmoXWV*_1062_ cluster from strain HTCC1062. As shown in Figure 3, the Δ*tmoW*_*DSS–3*_ mutant was unable to grow on TMAO, consistent with that previously reported (Lidbury et al., 2014). In contrast, the growth of the complemented strain Δ*tmoW*_*DSS–3*_-*tmoXWV*_1062_ on TMAO was comparable to that of strain *R. pomeroyi* DSS-3, suggesting that the *tmoXWV*_1062_ cluster was involved in TMAO transport in strain Δ*tmoW*_*DSS–3*_-*tmoXWV*_1062_. Considering that strain HTCC1062 can grow on TMAO (Figure 2A), this result indicates that *tmoXWV*_1062_ is most likely to encode a functional TMAO importer in strain HTCC1062.

### Characterization of TmoX_1062_

**FIGURE 4:**
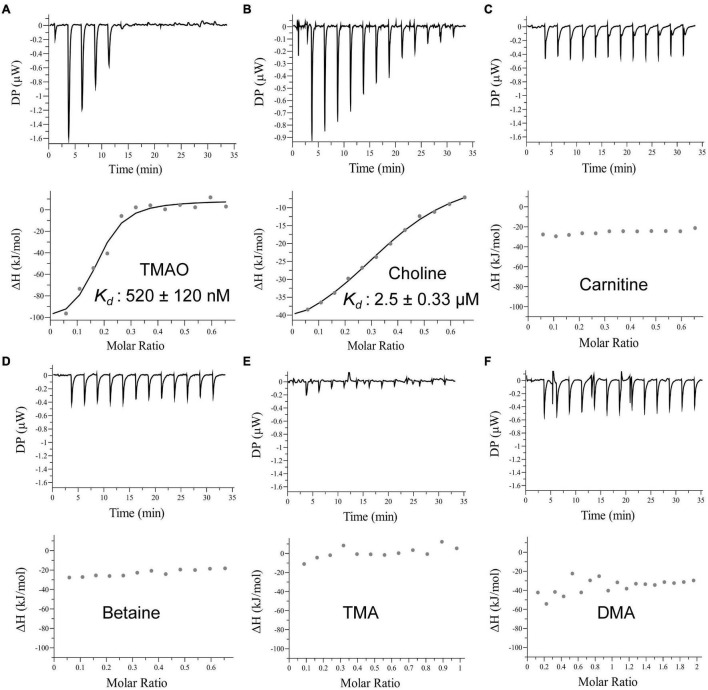
ITC data for titrations of different substrates into TmoX_1062_. ITC traces (top) and integrated binding isotherms (bottom) are shown. Substrates are shown in each integrated binding isotherm.

The periplasmic substrate binding protein of an ABC transporter is usually responsible for the first-step recognition of substrate, and can bind a given ligand with high affinity (Albers et al., 1999; Chen C. L. et al., 2010). To characterize TmoX_1062_, the substrate binding protein of TmoXWV_1062_, the full-length *tmoX*_1062_ gene containing 934 nucleotides was amplified from the genome of strain HTCC1062 and was expressed in *E. coli* BL21(DE3) cells. To analyze the substrate specificity of TmoX_1062_, the binding affinities of the recombinant TmoX_1062_ toward TMAO, betaine, choline, TMA, DMA and carnitine were determined by ITC measurements. Among the tested substrates, TmoX_1062_ possessed a high binding affinity toward TMAO, with a *K*_*d*_ (dissociation constant) of 520 nM (Figure 4A), but presented little binding affinity toward betaine, carnitine, TMA or DMA (Figure 4 and Table 1). Compared to TmoX_DSS–3_ of *R. pomeroyi* DSS-3, which exhibited a *K*_*d*_ of 1.6 μM toward TMAO (Li et al., 2015), TmoX_1062_ possessed a higher binding affinity toward TMAO. Considering the concentrations of TMAO range from nanomolar to low micromolar in marine environments (Gibb et al., 1999; Gibb and Hatton, 2004), the higher binding affinity of TmoX_1062_ toward TMAO would help strain HTCC1062 gain a competitive advantage over other bacteria at low TMAO concentrations. Surprisingly, the recombinant TmoX_1062_ also presented binding affinity toward choline, with a *K*_*d*_ of 2.5 μM (Figure 4B and Table 1). A similar phenomenon was also observed in TmoX_DSS–3_ (Li et al., 2015). RT-qPCR results indicated that choline did not induce the transcription of *tmoX*_1062_ in strain HTCC1062 (Supplementary Figure 2), suggesting that the binding of recombinant TmoX_1062_ toward choline may not make physiological sense. Alternatively, strain HTCC1062 may utilize TmoXWV_1062_ as a multifunctional transporter to import both TMAO and choline, as this strain possesses a highly streamlined genome (Giovannoni et al., 2005; Sun et al., 2011; Noell and Giovannoni, 2019).

**TABLE 1:** Thermodynamic parameters determined by ITC measurements.

Substrate	*K*_*d*_ (μM)	Δ*H* (kcal/mol)	−*T*Δ*S* (kcal/mol)
TMAO	0.52 ± 0.12	−117.0 ± 16.3	80.9
Betaine	–	–	–
Choline	2.5 ± 0.33	−48.7 ± 4.3	32
TMA	–	–	–
Carnitine	–	–	–
DMA	–	–	–

*–, little binding activity was detectable under the experimental conditions.*

**FIGURE 5:**
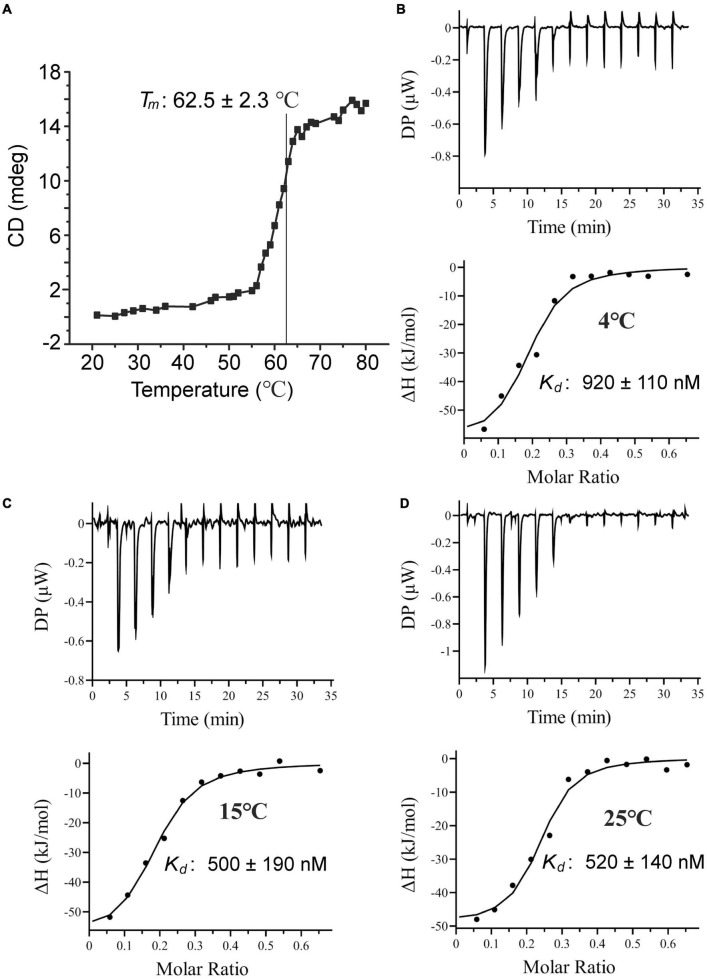
Characterization of TmoX_1062_. **(A)** The *T*_m_ of TmoX_1062_ determined by CD. **(B–D)** ITC data for titrations of TMAO into TmoX_1062_ at 4°C **(B)**, 10°C **(C)**, and 25°C **(D)**.

The *tmoX* gene is widespread in divergent marine bacteria, especially in SAR11 bacteria (Lidbury et al., 2014). The seawater temperatures are different at different depths and change with the seasons regularly, especially for surface seawaters (Malmstrom et al., 2010). Therefore, marine bacteria need to adapt different temperatures. To investigate the thermostability of TmoX_1062_, we measured the melting temperature (*T*_m_) of TmoX_1062_. The *T*_m_ of TmoX_1062_ is 62.5°C (Figure 5A), which is higher than that of TmoX_*DSS–3*_ (*T*_m_ = 54.5°C) (Li et al., 2015), suggesting that TmoX_1062_ has higher thermostability than TmoX_*DSS–3*_. The binding affinities of TmoX_1062_ toward TMAO at different temperatures were also detected. TmoX_1062_ exhibited high binding affinities toward TMAO at 4°C (Figure 5B), 10°C (Figure 5C) and 25°C (Figure 5D), indicating that TmoXWV_1062_ should be able to import TMAO into cells of strain HTCC1062 efficiently at different temperatures. The nanomolar-level TMAO binding affinity, the high thermostability and the strong temperature adaptability of TmoX_1062_ may reflect the niche adaptation of HTCC1062 to the volatile marine environment, especially to the oligotrophic environment.

### Key Residues of TmoX_1062_ Involved in Binding Trimethylamine *N*-Oxide

**FIGURE 6:**
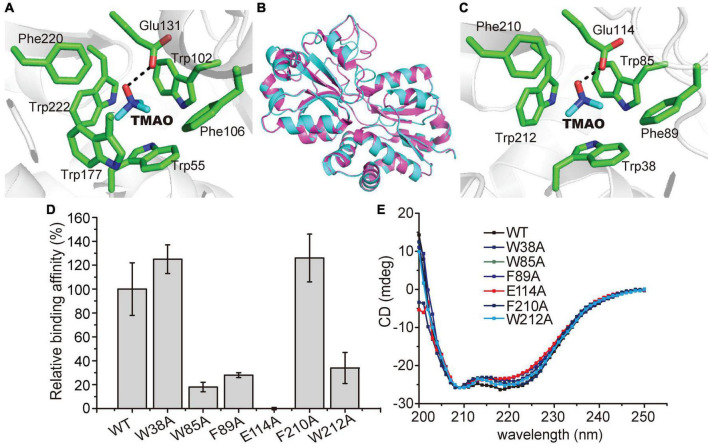
Possible residues involved in the binding of TMAO in TmoX_1062_. **(A)** Residues composing the TMAO binding pocket in TmoX_DSS–3_ (PDB code: 4XZ6). The TMAO molecule is colored in blue, and TmoX_DSS–3_ residues are colored in green. The possible hydrogen bond is represented by the dashed line. **(B)** Superimposed structures of TmoX_DSS–3_ (purple) and TmoX_1062_ (cyan). **(C)** Residues composing the TMAO binding pocket based on modeling structure of TmoX_1062_. The TMAO molecule is colored in blue, and TmoX_1062_ residues are colored in green. The possible hydrogen bond is represented by the dashed line. **(D)** Binding affinities of WT TmoX_1062_ and its mutants toward TMAO. The binding affinity of WT TmoX_1062_ was defined as 100%. The error bar represents standard deviation of triplicate experiments. **(E)** CD spectra of WT TmoX_1062_ and its mutants.

The TMAO binding mechanism of TmoX_DSS–3_ has been proposed based on structural and mutational analyses (Li et al., 2015). In TmoX_DSS–3_, the TMAO binding pocket is composed of Trp55, Trp102, Phe106, Glu131, Trp177, Phe220, and Trp222 (Figure 6A), among which Glu131 forms a hydrogen bond with the oxygen atom of TMAO, and four tryptophan residues (Trp55, Trp102, Trp177, and Trp222) form a rectangular aromatic box and interact with TMAO by cation-π interactions (Li et al., 2015; Figure 6A). The aromatic rings of two phenylalanine residues (Phe106 and Phe220) also participate in forming the hydrophobic cage to accommodate TMAO (Li et al., 2015; Figure 6A). TmoX_1062_ shares ∼51% sequence identity with TmoX_*DSS–3*_, and the TMAO binding mechanism of TmoX_1062_ is still unclear.

To probe the TMAO binding mechanism of TmoX_1062_, we tried to co-crystallize TmoX_1062_ and TMAO and solve the crystal structure of TmoX_1062_. However, all the attempts failed. We then modeled the structure of TmoX_1062_ *via* Swiss-model^2^ (Waterhouse et al., 2018), with the crystal structure of TmoX_DSS–3_ (PDB code: 4XZ6) as the template. The overall structure of TmoX_1062_ is similar to that of TmoX_*DSS–3*_ (Figure 6B), with a root mean square deviation (RMSD) between these two structures of 0.1 Å over 226 Cα atoms.

Structural analysis of the model of TmoX_1062_ showed that the binding pocket of TmoX_1062_ may be composed of Trp38, Trp85, Phe89, Glu114, Phe210, and Trp212 (Figure 6C), and Trp164, the corresponding residue of Trp177 in TmoX_DSS–3_ (Figure 6A), may not participate in binding TMAO. The residue Glu114, corresponding to Glu131 in TmoX_DSS–3_, may form a hydrogen bond with TMAO (Figure 6C). Mutation of Glu114 to alanine abolished the binding affinity of TmoX_1062_ toward TMAO (Figure 6D), indicating the important role of Glu114 in binding TMAO. The side chains of Trp38, Trp85 and Trp212 (corresponding to Trp55, Trp102 and Trp222 in TmoX_DSS–3_, respectively) form an aromatic box (Figure 6C). Together with the side chains of Phe89 and Phe210 (corresponding to Phe106 and Phe220 in TmoX_DSS–3_), this box forms a hydrophobic cage to accommodate the quaternary amine of TMAO (Figure 6C). Mutations of Trp85, Phe89 and Trp212 to alanine severely deceased the binding affinity of TmoX_1062_ toward TMAO, suggesting the important roles of these three residues in substrate binding (Figure 6D). However, mutants W38A and F210A still maintained a relatively high TMAO binding affinity (Figure 6D), indicating that these two residues may not participate in TMAO binding, or the other residues of TmoX_1062_ may compensate the function of Trp38 and Phe210. In TmoX_DSS–3_, mutations of the corresponding residues composing the TMAO binding pocket all decreased its TMAO binding affinity to a large extent (Li et al., 2015). Therefore, our biochemical results suggested that several residues of TmoX_1062_ participating in TMAO binding may be different from those of TmoX_*DSS–3*_, although sequence analysis showed that the residues comprising the binding pocket of TmoX are all highly conserved in the MRC and the SAR11 clade (Li et al., 2015). CD spectroscopy assays showed that the secondary structures of the mutants are similar to that of WT TmoX_1062_ (Figure 6E), indicating that the decrease in the binding affinities of the mutants is a result of residue replacement rather than structural changes.

## Conclusion

Trimethylamine *N*-oxide is widespread in the oceans, and can be utilized by marine bacteria as carbon, nitrogen and/or energy source (Lidbury et al., 2015, 2017). The SAR11 bacteria are widespread in marine environment (Brown et al., 2012). Here, our results showed that the SAR11 bacterium HTCC1062 is capable of utilizing TMAO as a nitrogen source for growth, which likely absorbs TMAO *via* the ABC transporter TmoXWV_1062_. The periplasmic substrate binding protein TmoX_1062_ of this transporter has high binding affinity toward TMAO, and exhibits a relatively high thermostability and strong temperature adaptability, which may reflect the niche adaptation of HTCC1062 to the oligotrophic marine environment. Mutational analysis indicated that the TMAO binding mechanism of TmoX_1062_ may have differences from the previously reported mechanism of TmoX_DSS–3_ of MRC bacteria. This study provides insights into how SAR11 bacteria utilize TMAO and offers a better understanding of marine nitrogen cycling.

## Data Availability Statement

The original contributions presented in the study are included in the article/Supplementary Material, further inquiries can be directed to the corresponding author/s.

## Author Contributions

C-YL and Y-ZZ designed the research. X-LC and J-MD directed the research. CG performed the experiments. NZ and X-YH helped in experiments. NW, X-YZ, and PW helped in data analysis. CG, C-YL, and X-LC wrote the manuscript. C-YL edited the manuscript. All authors contributed to the article and approved the submitted version.

## Conflict of Interest

The authors declare that the research was conducted in the absence of any commercial or financial relationships that could be construed as a potential conflict of interest.

## Publisher’s Note

All claims expressed in this article are solely those of the authors and do not necessarily represent those of their affiliated organizations, or those of the publisher, the editors and the reviewers. Any product that may be evaluated in this article, or claim that may be made by its manufacturer, is not guaranteed or endorsed by the publisher.

## Footnotes

1
http://www.dsmz.de/

2
https://swissmodel.expasy.org/interactive
