# Supplementary material for: Characterization of the Trimethylamine N-Oxide Transporter From Pelagibacter Strain HTCC1062 Reveals Its Oligotrophic Niche Adaption
Source: Front Microbiol. 2022 Feb 28;13:838608. doi: 10.3389/fmicb.2022.838608 (PMC8918994; doi:10.3389/fmicb.2022.838608)
Supplement: Supplementary file 1 [file Data_Sheet_1.DOCX]

Supplementary Material


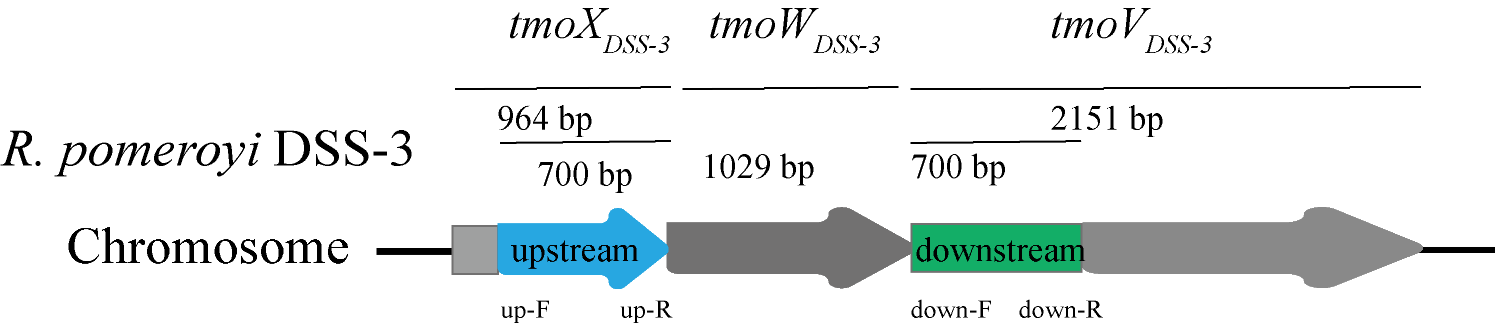


**FIGURE S1**. Construction of the *tmoW*-deleted mutant of *R. pomeroyi* DSS-3.





# FIGURE S2. RT-qPCR assay the transcriptions of *tmoX_1062_* in response to choline. The bacterium cultured without choline in the same medium was used as the control. The *recA* gene was used as an internal reference gene. The error bar represents standard deviation of triplicate experiments.

Table S1. Primers used in this study.

| **Primers** | **Sequence (5’-3’)** | **Purpose** |
| --- | --- | --- |
| RT-*tmoX_1062_*-F | TAGACGAATTGACTCATACAC | Used for RT-qPCR of the *tmoX_1062_* gene |
| RT-*tmoX_1062_*-R | TAACGCAGCAGACAGTAA |  |
| RT-*tdm_1062_*-F | ATGAATACAGTGGTGAATGG | Used for RT-qPCR of the *tdm_1062_* gene |
| RT-*tdm_1062_*-R | TGATTGATGGTTGGATTGG |  |
| RT-*gmaS_1062_*-F | GGTTAATGGATGGAGCAGTA | Used for RT-qPCR of the *gmaS_1062_* gene |
| RT-*gmaS_1062_*-R | GCATCTTCAATTATGTCAGGTAA |  |
| RT-*recA*-F | CACGAACACAATGATGATT | Used for RT-qPCR of the *recA* gene |
| RT-*recA*-R | GGCACCAATTCTTCTAATG |  |
| *tmoW_DSS-3_*-UP-F | GGGGACAAGTTTGTACAAAAAAGCAGGCTGCGTGACCACGACCGAGACG | Upstream fragment of the *tmoW_DSS-3_* gene |
| *tmoW_DSS-3_*-UP-R | CTTATTCAATAGAAATTATACAAGCGTTTATACATTATAT |  |
| *tmoW_DSS-3_*-Down-F | GAGCGCCTGACAGCGTTTGCCTGGATCCCCAGCACTAATCTTATGAC | Downstream fragment of the *tmoW_DSS-3_* gene |
| *tmoW_DSS-3_*-Down-R | GGGGACCACTTTGTACAAGAAAGCTGGGTGACCCCGAACGTGCCCCATTA |  |
| *tmoXWV_1062_*-350Up-F | TAAACCGGTGCTAGCCCCGGGTGGTACCTGAATTCTTATTCTCTCTCCCTTAATATTAAGTTAT | Complementation of the Δ*tmoW_DSS-3_* mutant |
| *tmoXWV_1062_*-Down-R | TCTATTGCTGGTTTACCGGTACTAGTAGGATCCGCCAATGCCCAGATGTTTTTTACGTTTGCCCACC |  |
| *tmoX_1062_*-F | AAGAAGGAGATATACATATGAAGAAGATCGTTAGTCTGATGAGTG | Amplification of the *tmoX_1062_* gene |
| *tmoX_1062_*-R | TGGTGGTGGTGGTGCTCGAGTTTGGTAAACGGGGTCCACAC |  |
